# Poisoning with Tainted Meat

**Published:** 1878-01-20

**Authors:** L. B. Anderson

**Affiliations:** Virginia


### *POISONING WITH TAINTED MEAT.*

By L. B. Anderson, M.D., of Virginia.

On the 14th of December, 1877, I was called to see a gentleman many miles from my home. Arriving at the place in the afternoon, after a ride of twenty-five miles, and intending to visit other patients, I accepted an invitation to take a lunch of "hog's head and turnips." Though, at best, an unsavory dish to me, I determined to partake of a few morsels to allay hunger, and enable me the better to undergo the additional ride of some thirty miles that afternoon. The house was kept by two old widow women, one of whom was suffering greatly from cystitis and retention of urine. The room into which I was ushered to take my meal was cold, the "head and turnips" and bread were cold, and, I being cold, made haste to complete my dinner as soon as possible.

I took two small portions of meat and bread without detecting anything unusual. The third morsel, however, was peculiarly distasteful, and I then examined more carefully, and discovered that the portion I had eaten was semi-putrid. I took a large draught of water and left the room. I should have taken an emetic at once, but had no idea that so small a portion could exert any deleterious influence either on my stomach or constitution; and, besides, I had, at that moment, received an urgent call to hasten fifteen miles away, and my patient required the immediate use of the catheter. I, therefore, hastily drew off the retained urine, gave the necessary directions, and left.

In less than half an hour, a most distressing sensation was experienced in my stomach, which increased in intensity until I felt as though the inner coat was being corroded by some active caustic. This sensation was followed by a giddy, swimmy feeling in the head, which rendered it difficult for me to retain my seat in the sulky. Passing the house of a friend, after traveling about thirteen miles, I felt so feeble and exhausted that I procured and drank a glass of wine. This allayed the distressing symptoms for awhile. After leaving the bedside of my patient, all the symptoms returned with decided aggravation, and I, with great difficulty, made my way home at a late hour of the night. Having nothing in my stomach, I drank a glass of cold cream, and ate a few corn-meal batter-cakes. Thus filled, my stomach was very much relieved.

Being fatigued, I retired early, and soon fell asleep. My sleep, however, was dreamy and unrefreshing. Being in no way relieved, and having cases of great importance to attend to that day, I ate a breakfast similar to my supper. Again I felt relieved. About noon, however, the symptoms became very distressing, and I hastened homeward. When I entered my chamber, my stomach felt like it was being consumed by a pent-up fire, while my head was reeling and my mind greatly confused. I called for a pitcher of warm water, and got into bed. As soon as the water came, I drank as much of it as I could possibly swallow. I soon commenced vomiting a most rancid and offensive fluid, some of which looked like pus. After free emesis, I was so much better that I went to see some diphtheritic patients four miles off. On my return, I felt as badly as at any previous time. I then took an active vegetable cathartic, which operated copiously before the next morning.

I experienced but little relief from these means, and I really could not spare the time to use other remedies. During that day, my whole system was in an agony—swimming of the head, burning at the precordia, a sense of sinking, attended with involuntary sighing, pains in the heart, constriction across the chest, a dreamy, weary state of mind, which was exceedingly exhausting and prostrating. Never before, in all the long catalogue of physical and mental sufferings I had undergone, had I experienced such indescribable symptoms. My mental seemed to have separated from my physical being, and was in a dreamy, obscure, restless, hazy state, without support, and struggling in vain for a moment's repose. The difficulty of inhalation rapidly increased, and I felt that life would be sustained but a short time.

I took a teaspoonful of citrate of potassium, and five grains of salicylic acid, in half a glass of water, and wrapped up in bed. My pulse was feeble and somewhat hurried; my heart was laboring, with occasional stitches of pain; my respiration was almost entirely a succession of sighs, while my feet and hands were cold, my flesh bathed in a cold perspiration, and slight rigors frequently agitating my muscles. So great was the oppression, and sense of sinking, that I took another glass of wine. In a short time after swallowing these agents, I began to eructate large volumes of gas, accompanied with a most gratifying sense of relief.

I continued to use the salicylic acid, occasionally, for several days, restricted my diet to the blandest articles, and was compelled to abstain from the use of tobacco, which I found to produce a most uncomfortable sense of oppression and nervous prostration.

Ten days have elapsed since I ate the three small morsels of tainted meat, and though I am greatly improved, I still feel the unpleasant sensations above described, in my head, heart, lungs, and stomach, in a greatly modified degree.

The salicylic acid has always produced free eructations soon after taking it, affording marked relief, while nothing I have ever before used has aroused to such activity the urinary secretion. While one might infer from its taste that it is an active astringent, it has exerted no constipating effect upon my bowels whatever.

